# Comparisons of the Pentax-AWS, Glidescope, and Macintosh Laryngoscopes for Intubation Performance during Mechanical Chest Compressions in Left Lateral Tilt: A Randomized Simulation Study of Maternal Cardiopulmonary Resuscitation

**DOI:** 10.1155/2015/975649

**Published:** 2015-06-16

**Authors:** Sanghyun Lee, Wonhee Kim, Hyunggoo Kang, Jaehoon Oh, Tae Ho Lim, Yoonjae Lee, Changsun Kim, Jun Hwi Cho

**Affiliations:** ^1^Department of Emergency Medicine, College of Medicine, Hanyang University, Seoul 133-792, Republic of Korea; ^2^Department of Emergency Medicine, College of Medicine, Hallym University, Seoul, Republic of Korea; ^3^Department of Emergency Medicine, Kangwon National University School of Medicine, Chuncheon, Republic of Korea

## Abstract

*Purpose*. Rapid advanced airway management is important in maternal cardiopulmonary resuscitation (CPR). This study aimed to compare intubation performances among Pentax-AWS (AWS), Glidescope (GVL), and Macintosh laryngoscope (MCL) during mechanical chest compression in 15° and 30° left lateral tilt. *Methods*. In 19 emergency physicians, a prospective randomized crossover study was conducted to examine the three laryngoscopes. Primary outcomes were the intubation time and the success rate for intubation. *Results*. The median intubation time using AWS was shorter than that of GVL and MCL in both tilt degrees. The time to visualize the glottic view in GVL and AWS was significantly lower than that of MCL (all *P* < 0.05), whereas there was no significant difference between the two video laryngoscopes (in 15° tilt, *P* = 1; in 30° tilt, *P* = 0.71). The progression of tracheal tube using AWS was faster than that of MCL and GVL in both degrees (all *P* < 0.001). Intubations using AWS and GVL showed higher success rate than that of Macintosh laryngoscopes. *Conclusions*. The AWS could be an appropriate laryngoscope for airway management of pregnant women in tilt CPR considering intubation time and success rate.

## 1. Introduction

Emergency physicians are primarily responsible for both high-quality chest compressions and rapid advanced airway management in cardiopulmonary resuscitation (CPR) for women who are both pregnant and nonpregnant. The 2010 European Resuscitation Council (ERC) guidelines recommend employing manual displacement of the uterus with left lateral tilt (LLT) to achieve aortocaval decompression during CPR in late pregnancy [1]. High-quality chest compressions could be performed in the LLT position, which is not flat in simulated manikin studies [2]. Mechanical chest compression devices could compress the chest sufficiently regardless of the tilt [2, 3]. Intubation time and the success rate for intubation are influenced by manual chest compressions and the type of laryngoscope [4–6]. The LLT position could also interfere with rapid intubation during chest compressions in the operating setting [7, 8]. Physiological changes in a pregnant woman's airway (i.e., tongue swelling and airway edema) make endotracheal intubation more difficult [9–11]. Decreased functional residual capacity and increased risk of aspiration in a pregnant woman require rapid endotracheal intubation during CPR [12–14].

The optimal degree of LLT is not known. However, ERC guidelines recommend it be between 15° and 30° [1]. In an emergency room or delivery room, where maternal cardiac arrest commonly occurs, several methods are available to achieve the LLT position, whereas the operating table is used in the operating room [15–17]. However, only a foam and hard wedge can maintain an angle of 15°–30° [18, 19]. No study has investigated the optimal laryngoscope to be used for rapid and successful intubation during chest compressions in maternal CPR using a mechanical compression device with an angle of 15° and 30° in the LLT position in an emergency room setting.

The aim of this study was to evaluate laryngoscopes for intubation on simulated difficult airways during maternal CPR with a mechanical compression device at angles of 15° and 30° in the LLT position in an emergency room setting. We hypothesized that the required time and success rate for intubation would be different based on the type of laryngoscope used in the above-mentioned situation.

## 2. Methods

### 2.1. Study Design

We conducted a randomized crossover manikin study to examine intubation with three laryngoscopes under two types of tilt during simulated maternal CPR at our university's simulation centre in March, 2014. The local ethics committee approved this study in January 2014 (HYI-14-004-1). We registered the study protocol in Clinical Trials before study initiation (Clinicaltrials.gov: NCT02074072).

### 2.2. Equipment and Materials

**Figure 1:**
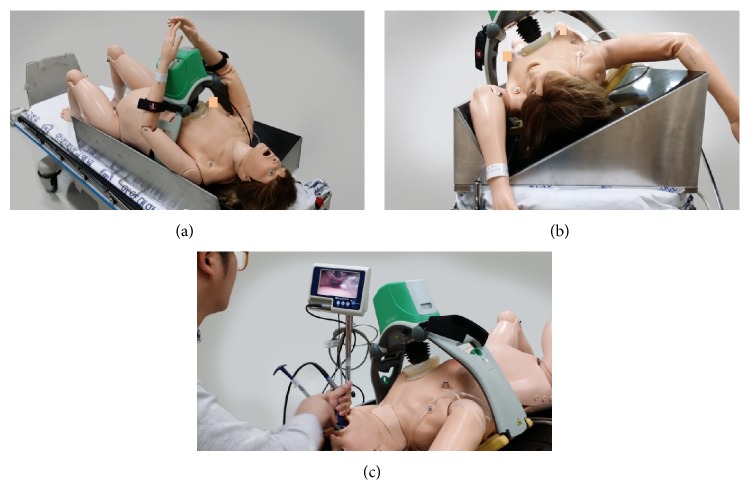
Endotracheal intubation with the Glidescope during mechanical chest compressions by LUCAS on the wedge. (a) High-fidelity manikin (SimMom) applying LUCAS on the 15° wedge. (b) 30° left lateral tilt position created by custom-made wedge. (c) Trial scene with Glidescope during mechanical chest compressions in the 30° wedge.

Participants intubated the airway with a direct laryngoscope and two video laryngoscopes using an endotracheal tube with an internal diameter of 6.5 mm (Portex, St. Paul, MN, USA) and the manufacturer stylet. A direct laryngoscope is the Macintosh laryngoscope (MCL), which has a size-4 curved blade with a Satin Slip Stylet (Mallinckrodt Medical, St. Louis, MO, USA). One video laryngoscope option is the Glidescope (GVL), which has a hyperangulated, nonchannelled, standard-size blade (Verathon, Bothell, WA, USA) with a GlideRite Rigid Stylet. Another option is the Pentax-AWS (AWS), which has a channelled, standard blade (Pentax Corporation, Tokyo, Japan). We used a high-fidelity manikin (SimMom, Laerdal, Stavanger, Norway) for chest compressions and airway intubations. The manikin was set with a tongue edema setting and LUCAS 2 (LUCAS Chest Compression System, Physio-Control/Jolife AB, Lund, Sweden) for performing chest compressions at a continuous rate of 100 compressions/min at a depth of 5 cm, according to guidelines [1]. We used 15° and 30° custom-made wedges to simulate the LLT position (1000 × 600 × 156 mm for the 15° wedge and 1000 × 600 × 300 mm for the 30° wedge). The manikin was laid on a backboard (450 × 600 × 10 mm, 3 kg Lifeline Plastic, Sung Shim Medical Co., Bucheon, Korea) and placed on the wedge on a bed (Transport stretcher, 760 × 2110 mm, 228 kg, Stryker Co., Kalamazoo, Michigan, US) (Figure 1).

### 2.3. Participants

The sample size was calculated based on a previous study regarding the time required for intubation during chest compressions with a 27° LLT [7]. The mean (SD) time was MCL 18.9(4) and AWS 12.6(1.2) to ventilate the lungs after tracheal intubation. To detect a 33% difference in intubation time with a power of 0.8, we estimated that 14 operators would be adequate for each device with a 20% drop rate. We recruited physicians working at one tertiary medical centre in March, 2014. We included healthy volunteers who were between 16 and 60 years old and had more than 50 experiences of intubation using MCL [20]. We excluded people who had wrist and low back disease. All participants signed a written consent form before being included.

### 2.4. Interventions

**Figure 2:**
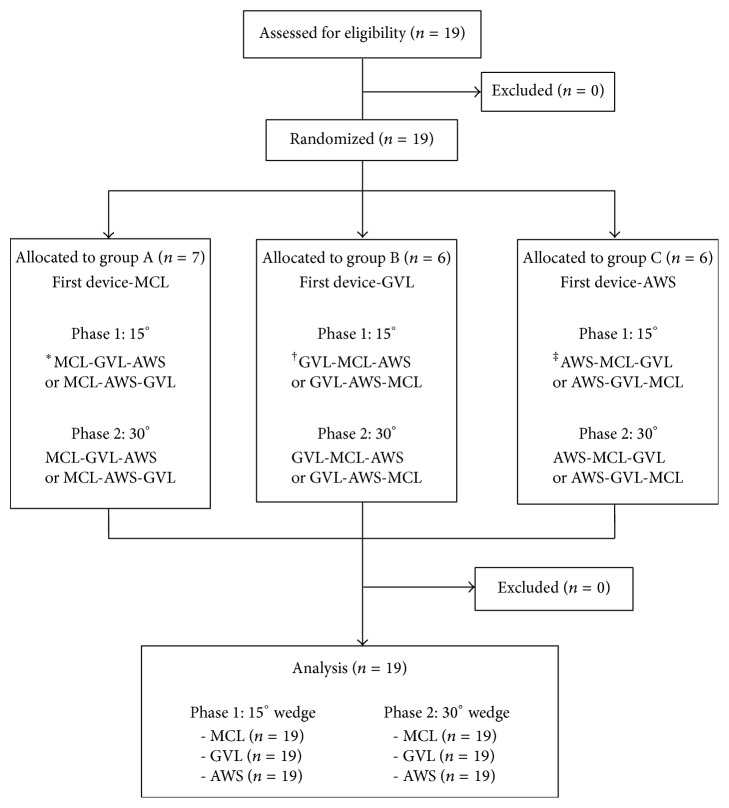
Diagram showing the flow of participants through the study.  ^*^MCL = Macintosh laryngoscope;  ^†^GVL = Glidescope;  ^‡^AWS = Pentax-AWS.

All participants completed a brief questionnaire consisting of demographic information (age, gender, body weight, and height) and prior experiences of intubations and maternal CPR in a clinical situation. Ten minutes prior to starting the trials, participants were allowed to practice intubations with all laryngoscopes to familiarize themselves with the Laerdal Airway Management Trainer (Laerdal, Stavanger, Norway) without chest compressions and tongue edema under supine position. Nineteen participants were enrolled, and they were randomly allocated to three groups (http://www.random.org/). Participants in group A (*n* = 7) performed the first intubation with MCL during chest compressions under simulated 15° and 30° LLT positions, whereas participants in group B (*n* = 6) performed the first intubation with GVL. Group C (*n* = 6) performed the first intubation with AWS. After being allocated to the three groups, the participants were placed in a random order by a computer-generated list of random numbers (http://www.random.org/) to minimize learning effects, and then they performed intubation with the laryngoscopes (Figure 2). For MCL, the manikin's head and neck were placed in a sniffing position utilizing several rolled sheets. When each participant performed intubation with each laryngoscope, continuous chest compressions were performed by LUCAS 2. The height of the bed was 80 cm, which was approximately the height of the participant's mid-thigh level. Participants had a 10-minute break after each intubation in one LLT position and a 30-minute break before.

### 2.5. Outcomes

Primary outcomes were the intubation time and the success rate for intubation. The intubation time was recorded from the start-point to the mid-point and from the mid-point to the endpoint by a recorder. The recorder was informed about how to record the intubation time and was blinded to the objective of this study. The start-point was when the participant inserted the blade between the teeth after command to start by a recorder. The mid-point was when the participant exposed the vocal cord and stated “I can see.” The endpoint was at the first manual ventilation after intubation, regardless of success or failure of air inflating into the manikin's lungs. The time to visualize the glottis view (TTV) was measured from the start-point to the mid-point, and the time to progress the endotracheal tube (TTP) was consecutively measured from the mid-point to the endpoint. The time to intubate (TTI) was calculated from the start-point to the endpoint (TTV + TTP). We defined intubation failure as follows: when the tip of the tube is not properly placed in the trachea but is placed in the oesophagus or in the oral cavity or when the TTI is 90 sec or more [21, 22].

Secondary outcomes were the glottic view using a Cormack-Lehane score (CLS) and the preference for laryngoscopes. The preference for laryngoscopes was determined by asking the participants to choose the laryngoscope that would be most favourable during maternal CPR.

### 2.6. Statistical Analysis

The data were compiled using a standard spreadsheet application (Excel, Microsoft, Redmond, WA, USA) and were analysed using the Statistical Package for the Social Sciences (SPSS) 18.0 KO for Windows (SPSS Inc., Chicago, IL, USA). We generated the descriptive statistics and presented them as frequencies and percentages for the categorical data and medians with interquartile ranges (IQR) for the continuous data because the data were not normally distributed. To compare the intubation time among the three laryngoscopes, the Kruskall-Wallis test was used for continuous variables. A *χ* ^2^ test was used to compare the categorical variables, such as the success rate for intubation, the CLS, and the laryngoscope preference. A post hoc analysis was conducted with the Mann-Whitney test using a Bonferroni correction. *P* < 0.05 was considered significant. The Kaplan-Meier analysis was performed to analyse the cumulative success rate regarding TTV and TTP.

## 3. Results

### 3.1. General Characteristics

Nineteen participants were enrolled. Nineteen intubation trials were performed for each laryngoscope for both 15° and 30° LLT. There was no exclusion in our study. The general characteristics of the participants are shown in Table 1.

### 3.2. Tracheal Intubation in 15° LLT Position

TTI was significantly different among the three laryngoscopes. The TTI in AWS was shortest, followed by GVL and MCL (all *P* < 0.05). The TTV in both GVL and AWS was significantly less than that of MCL, whereas there was no significant difference between the two video laryngoscopes (*P* = 1.00). In terms of TTP, progression of ETT using AWS was faster than that of MCL and GVL (all *P* < 0.001). However, there was no significant difference between MCL and GVL (*P* = 0.56) (Table 2).

Intubation using AWS showed the highest success rate, followed by GVL and MCL. The glottis views in the two video laryngoscopes were better than that of MCL.

### 3.3. Tracheal Intubation in 30° LLT Positions

The TTI in AWS was faster than that of MCL and GVL (all *P* < 0.05). However, there was no significant difference between MCL and GVL (*P* = 0.08). The TTV in GVL and AWS was significantly lower than that of MCL, whereas there was no significant difference between the two video laryngoscopes (*P* = 0.71). In terms of TTP, progression of ETT using AWS was faster than that of MCL and GVL (*P* = 0.002, *P* < 0.001, resp.). However, there was no significant difference between MCL and GVL (*P* = 0.55) (Table 3).

**Figure 3:**
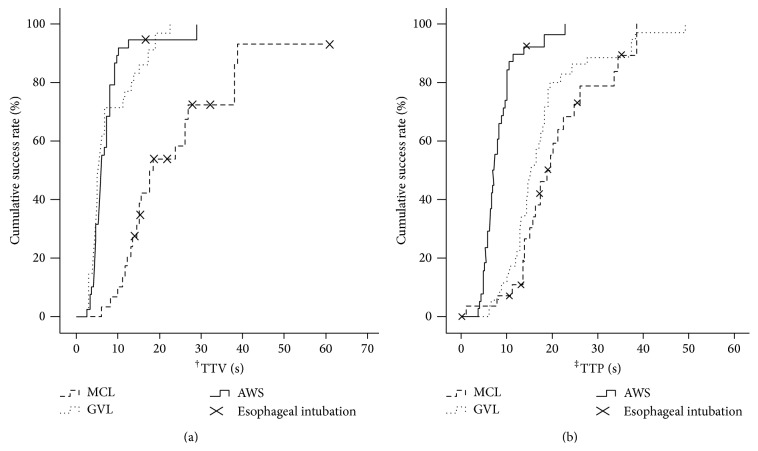
Cumulative success rate related to (a) the time from blade insertion to exposure of the vocal cords (TTV) and (b) the time from exposure of the vocal cords to manual ventilation (TTP) in the left lateral tilt position with mechanical chest compressions. MCL: Macintosh laryngoscope; AWS: Pentax-AWS; GVL: Glidescope; ^†^TTV: time to visualize glottis view; ^‡^TTV: time to progress the tracheal tube from exposure of vocal cords to first ventilation.

The cumulative success rates related to TTV for AWS and GVL were significantly higher than that of MCL (*P* < 0.001). The cumulative success rate related to TTP for AWS was significantly higher than that of GVL and MCL (*P* < 0.001) (Figure 3).

### 3.4. Preference for Laryngoscopes

Regardless of tilt degree, 13 participants (68.4%) preferred AWS, and six participants (31.6%) selected GVL when asked which of the three laryngoscopes they prefer for maternal CPR.

## 4. Discussion

Pregnancy can be a potent contributor to ventilation difficulty because of edema of the upper airway tract, and motion limitation of the thorax and diaphragm may occur unexpectedly [9–11]. Supraglottic airway devices might be useful to protect the airway and provide ventilation during pregnancy. However, it is known that aspiration is a high risk factor for pregnant women [1]. Compared to an endotracheal tube (ETT), supraglottic airway devices are not enough to prevent aspiration. Therefore, emergent endotracheal intubation in pregnant women deserves special emphasis [1, 11].

It can be difficult to perform intubation during CPR because the chest compressions can cause the glottis to move up and down, which makes it hard to maintain proper glottis view [7]. Furthermore, because patients are placed in the LLT position during maternal CPR, the lifting force of the intubator to expose the glottis view can be insufficiently transmitted to patients. It is also difficult to handle the laryngoscope with patients in the LLT position [7]. Even if the glottis is fully exposed, chest compressions and the LLT position can interrupt intubators in the progression of the ETT and removal of the stylet [7]. Therefore, the TTI can be affected by the degree of tilt, chest compression, and the type of laryngoscope. Kohama et al. reported TTI in AWS during simulated maternal CPR (normal airway setting, manual chest compression, and 27° LLT position) using an operating table that was shorter than that of MCL [7]. In this study, we evaluated the TTI for laryngoscopes in different settings (tongue edema setting, mechanical chest compression, and two types of LLT positions using 15° and 30° wedges) on a hospital bed in an emergency room and compared it to previous studies. We found that the use of AWS for emergent intubation reduced the TTV and TTP, which resulted in the reduction of the TTI compared with that of MCL at both tilt degrees. The use of GVL significantly decreased the TTV but hardly reduced the TTP, which also resulted in a reduction of the TTI compared with that of MCL. This means that the use of a camera attached to the blade of a video laryngoscope may play a major role in decreasing the time to exposure of the vocal cords. In terms of TTP, AWS showed better outcomes than GVL when compared with MCL. It is believed that the channelled blade applied to AWS enables the ETT to progress effectively [4]. Because AWS does not require a stylet, AWS can also reduce the time required to handle a stylet in GVL during maternal CPR. AWS was the most preferred device among the three laryngoscopes in this study, which we assume is due to the integrated channel of the blade that enables faster tracheal intubation without a stylet.

The ERC guideline recommends a 15°–30° LLT position to remove the caval compression during maternal CPR [1]. Two studies have reported that the TTI was affected by the patient position and the type of laryngoscope used. When using MCL, the TTI in the supine position was shorter than that in the LLT position [8]. However, in terms of AWS, there was no difference in the TTI between these positions [8]. In this study, participants performed intubations at two tilt angles (15° and 30°), excluding the supine position. Angles of 15° and 30° are the minimum and maximum tilt angles, respectively, that are recommended by the ERC guideline for maternal CPR [1]. There were no differences in the TTI between the tilt angles for the three laryngoscopes.

Regarding the success rate for intubation, previous studies have reported that this is affected by chest compressions and the type of laryngoscope used [7]. In the 27° LLT position, MCL showed a lower success rate with chest compressions than without chest compressions [7]. However, AWS showed an equal success rate regardless of chest compression [7]. In the supine position during chest compressions, MCL showed a lower success rate than AWS and GVL [4, 23]. In this study, we evaluated intubation performance in the 15° and 30° LLT positions during chest compression. AWS and GVL showed better success rates than MCL at both tilt degrees, as expected. The cumulative success rates had the same results.

## 5. Limitation

There were several limitations of this study.

We used a high-fidelity manikin setting with tongue edema to simulate a difficult airway during pregnancy. This manikin can also simulate difficult airway applying trismus setting, however, which has not been known as typical change of maternal airway. However, the actual maternal airway cannot be simulated by only tongue edema. Furthermore, there could be various clinical anatomic changes due to pregnancy in addition to tongue edema. Therefore, the measured intubation performance in this study could differ from that of actual maternal CPR. Further studies using more sophisticated manikin or clinical trials would be needed.

Recently, mechanical chest compression devices have been widely used, and some studies have reported that mechanical chest compression devices could show better outcomes than manual chest compressions [3]. We used LUCAS Chest Compression System (Physio-Control/Jolife AB, Lund, Sweden) for even and uninterrupted chest compressions. The effect of mechanical chest compressions on performing intubation during chest compressions might differ from that of manual chest compressions.

The most appropriate bed height for chest compressions is different from that of intubation. To best perform chest compressions, the bed height needs to be at lower- to mid-thigh level [24, 25]. In contrast, the bed height has to be raised to sternum level to perform intubation well. When intubation is performed using MCL, intubation performance could be influenced by the height of the bed. This is because the sightline view of the intubator is equated to the glottic opening to successfully intubate using MCL. However, in this study, the bed height was fixed at 80 cm, which was approximately mid-thigh level for the participants performing chest compressions. Therefore, the effects of bed height changes on intubation performance were not reflected in the study.

The pressures generated by laryngoscopes can cause deleterious effects to the soft tissues of the upper airway. If capillaries of airway soft tissue are engorged due to pregnancy, it can easily bleed under the pressure generated by laryngoscopes. In previous study, intubation using GVL could reduce the forces to the soft tissues of upper airway when compared to that of MCL [26]. Likewise, video laryngoscopes are considered to be advantageous to lower the pressure to the soft tissue of upper airway in maternal airway management. However, the pressures generated by laryngoscopes are not considered in this study.

## 6. Conclusions

The AWS could be an appropriate laryngoscope for airway management of pregnant women in tilt CPR considering intubation time and success rate.

## Figures and Tables

**Table 1:** Demographic characteristics.

Characteristics	Data
Sex (percent)	Male (100)
Age (years)	33 (29–36)
Height (cm)	172 (171–174)
Weight (kg)	78 (75–80)
BMI (kg/m^2^)	25.9 (24.2–26.6)
Participants	
PGY2^*^	3 (15.8)
PGY3	3 (15.8)
PGY4	2 (10.5)
EP^†^	11 (57.9)
Intubation experiences	
MCL > 50 (times)	19 (100)
GVL > 50 (times)	18 (94.7)
AWS > 50 (times)	5 (26.3)
Clinical experiences	
Maternal CPR	9 (47.4)
Maternal intubation	7 (36.8)
Intubation during maternal CPR	5 (26.3)

Categorical variables are given as numbers (percentage). Continuous variables are given as median (IQR). ^*^PGY: postgraduate years; ^†^EP: emergency physician.

**Table 2:** Tracheal intubation in 15-degree left lateral tilt positions (*n* = 19).

	15 degrees						
	MCL (*n* = 19)	GVL (*n* = 19)	AWS (*n* = 19)	*P* value	MCL versus AWS	MAC versus GVL	AWS versus GVL
Intubation time (seconds)							
TTI^*^	36.9	22.9	12.8	<0.001	<0.001	0.04	<0.001
	(31.5–50.7)	(17.5–33)	(11.3–15.2)				
TTV^†^	16.5	4.9	5.8	<0.001	<0.001	<0.001	1.00
	(12.8–26.3)	(4.2–13.5)	(4–7.6)				
TTP^‡^	18	16.2	7.0	<0.001	<0.001	0.56	<0.001
	(13.8–28)	(12.7–23.2)	(5.3–9.2)				
Success rate (percent)							
Success rate	14 (73.7)	18 (94.7)	19 (100)	0.04			
Failure							
Esophageal intubation	3 (15.8)	0 (0)	0 (0)	0.09			
Intubation time >90 s	2 (10.5)	1 (5.3)	0 (0)	0.77			
Cormack and Lehane score							
I-II	7 (36.8)	17 (89.5)	19 (100)	<0.001			
III-IV	12 (63.2)	2 (10.5)	0 (0)				
Preference	0 (0)	6 (31.6)	13 (68.4)	1.00			

Categorical variables are given as numbers (percentage). Continuous variables are given as median (IQR). MCL: Macintosh laryngoscope; AWS: Pentax-AWS; GVL: Glidescope. ^*^Total time for tracheal intubation. ^†^Time to visualize glottis view. ^‡^Time to progress the tracheal tube from exposure of vocal cords to first ventilation.

**Table 3:** Tracheal intubation in 30-degree left lateral tilt positions (*n* = 19).

	30 degrees						
	MCL (*n* = 19)	GVL (*n* = 19)	AWS (*n* = 19)	*P* value	MCL versus AWS	MCL versus GVL	AWS versus GVL
Intubation time (seconds)							
TTI^*^	32.4 (22.6–47.1)	21 (16.2–31.2)	13.4 (12.4–16.2)	<0.001	<0.001	0.09	0.001
TTV^†^	13.9 (9–23.9)	5.2 (4.0–8.9)	5.4 (4.7–8.2)	<0.001	<0.001	0.004	0.71
TTP^‡^	16.2 (13.6–21.8)	14.6 (12.3–18.6)	7.5 (6.3–9.9)	0.002	0.002	0.55	<0.001
Success rate (percent)							
Success rate	9 (47.4)	17 (89.5)	18 (94.7)	0.001			
Failure							
Esophageal intubation	4 (21.1)	0 (0)	1 (5.3)	0.11			
Intubation time >90 s	7 (36.8)	2 (10.5)	0 (0)	0.007			
Cormack-Lehane score							
I-II	5 (26.3)	18 (94.7)	19 (100)	<0.001			
III-IV	14 (73.7)	1 (5.3)	0 (0)				
Preference	0 (0)	6 (31.6)	13 (68.4)	1.00			

Categorical variables are given as numbers (percentage). Continuous variables are given as median (IQR). MCL: Macintosh laryngoscope; AWS: Pentax-AWS; GLS: Glidescope. ^*^Total time for tracheal intubation. ^†^Time to visualize glottis view. ^‡^Time to progress the tracheal tube from exposure of vocal cords to first ventilation.
